# Comprehensive Assessment of Tailing Dumps’ Impact on Water Quality of Rivers, Lakes, and Wells from Mining Areas

**DOI:** 10.3390/ijerph192214866

**Published:** 2022-11-11

**Authors:** Ovidiu Murarescu, Cristiana Radulescu, Ioana Daniela Dulama, George Muratoreanu, Gica Pehoiu, Raluca Maria Stirbescu, Ioan Alin Bucurica, Sorina Geanina Stanescu, Constantin Aurelian Ionescu, Andreea Laura Banica

**Affiliations:** 1 Faculty of Humanities, Valahia University of Targoviste, 130105 Targoviste, Romania; 2 Institute of Multidisciplinary Research for Science and Technology, Valahia University of Targoviste, 130004 Targoviste, Romania; 3 Faculty of Sciences and Arts, Valahia University of Targoviste, 130004 Targoviste, Romania; 4 Doctoral School Chemical Engineering and Biotechnology, Politehnica University of Bucharest, 060042 Bucharest, Romania

**Keywords:** mining, tailing dumps, uranium, copper, charcoal, contaminant, river, lake, well, health risk, carcinogenic risk, multivariate analysis

## Abstract

This study is the third in a series of investigations conducted by the authors, and certainly the most comprehensive research regarding the former uranium, copper, and charcoal mines from a particular geographical area of Romania. In this respect, the present scientific incursion focused on two areas containing former extraction uranium ore sites, Ciudanovita and Lisava, as well as copper ore from Moldova Noua and charcoal mines from Anina, Banat Region, Romania. It highlighted that, for the first time, the heavy metal concentration was correlated with the values of physicochemical indicators of water (i.e., EC, DO, pH, resistivity, salinity, and ORP), by using multivariate analysis, to shape a regional based model on spatial distributions and the variability of toxic contaminants from the hydrographic basin of Banat, Romania, as a consequence of former uranium, copper, and charcoal mines. In this regard, 11 metals including Al, Cr, Mn, Fe, Ni, Co, Cu, Zn, Sr, Cd, and Pb from different water samples (well, spring, river, and lake), collected from three mining areas (uranium, copper, and coal mines) were investigated. Non-carcinogenic and carcinogenic health risks of seven heavy metals were assessed using the EDI, DIM, and THQ. The obtained THQ values were within the acceptable limits for cancer risks for adults, but as regards children, eight samples out of 18 proved toxic. However, the HRI and THQ average values for Cd (0.265 adults/0.996 children) and Pb (0.025 adults/0.095 children) for children were 3–4 times higher than those for adults. This is a source of concern as their prevalence in well water exposes children and residents in the Banat Region to the risk of various types of cancers.

## 1. Introduction

In the last century, anthropogenic activities and major climate changes have led to the irreversible deterioration of surface water, on one hand, and of groundwater, on the other hand. Assuring good water quality is considered to be determinant for biodiversity. Particular attention should be given to freshwater resources, which usually satisfy the worldwide demand of people for drinking water. Waterborne diseases have frequently decimated the population of many cities due to chemical pollution [1,2,3]. Throughout the last decade, several chemical contaminants (i.e., anthropogenic source), often labelled as Contaminants of Emerging Concern (CECs), such as perfluorinated compounds (PFCs), alkylphenols, pharmaceuticals and personal care products (PPCPs), pesticides, carcinogenic metals (Cd, Ni, Cr, and As), present in water have come to the attention of authorities in terms of environmental and human health risk. Uncertainties over the occurrence, origin, and hazard of CECs and the large number of all kinds of emerging pollutants have prompted authorities to take measures and impose a set of priorities concerning the maximum accepted concentrations for these environmental contaminants [4,5,6,7]. Discovering the sources, and understanding the interactions and effects of water pollutants is essential for monitoring contaminants in a safe environment, and in an economically acceptable manner. There are two types of water contamination [8]: (1) permanent contamination that includes residues from industrial and mining activities, municipal waste, fertilizers, and pesticide spills from agricultural areas; and (2) occasional contamination, which includes transport accidents, toxic or radioactive spills. There is also a real risk potential for the contamination of groundwater with chemical waste from landfills, tailing dumps, treatment areas, and other facilities [9,10,11,12].

The monitoring process of chemical contaminants continues to concern the scientific world as this activity helps comprehend and predict the long-term health effects of chronic exposure to low concentrations of various pollutants, including heavy metals [9,10,13,14,15]. Most of the toxic metals are cancer-inducing agents according to the International Agency for Research on Cancer (IARC) [3,16]. Arsenic, cadmium, chromium, and nickel, in particular, are classified as group 1 carcinogens by the WHO [16]. Metal toxicity is modified by the action of environmental factors such as light, temperature and pH (e.g., soil or water). The removal procedure of these metals from water has a special significance since heavy metals are non-biodegradable and have long persistence in the environment, posing a threat to the biota due to their toxic effects [8,17,18,19,20]. On the other hand, the migration of metal ions into both surface waters and groundwater may occur and may be a threat to human health if the total content of metals exceeds the EU recommended exposure limits [3,21,22]. The interaction of heavy metal traces with organic compounds in water is extensive and was highlighted by various studies [23,24,25,26,27,28]. Overall, the interaction between heavy metals led to the development and assessment of risk-based approaches by the scientific world. In addition, one may also mention the interactions of metal-organic substances that involve organic species, such as ethylenediaminetetraacetic acid (EDTA), as a metal-chelating reagent, or organic matters (i.e., fulvic acid), respectively. All mentioned interactions are influenced by various factors, such as redox equilibrium, the formation-dissolution of precipitate, the formation and stability of colloid, acid-base balance, and microorganism-mediated reactions in water. Metal-organic interactions can increase or decrease the toxicity of metals in aquatic ecosystems and have a strong influence on the growth of algae in natural water [24,29,30]. In addition, mineral salts dissolved in water are good electricity conductors, while organic materials and colloids show poor electrical conductivity (EC). Therefore, the salinity and EC are significant quality indicators for using surface water for domestic purposes. Regarding the presence and hazard of heavy metals, it can be said that these metals are released into rivers, lakes, and wells due to various mining activities.

Bell and Donnelly [31] revealed that mining, known as one of the largest extraction industries in the world, is also one of the most important generators of residual waste and by-products resulted from extraction and processing. Rybicka highlighted that mining activity produces significant pollution of the atmosphere, soil, and waters with further impacts in the hydrogeological system, which eventually may lead to severe contaminations, reduced biodiversity, changes to the ecosystem, and soil stratigraphy [32]. On the other hand, the waste dumping generated by mining must be carefully monitored by authorities from each country in order to minimize environmental degradation and, consequently, their harmful impact on the quality of life [33,34]. In this respect, the tailing dumps generated by coal mining are associated with acidic and metal-rich drainage, noted as Acid Mine Drainage (AMD) [35,36], the consequences being a pH lower than 2.0, as well as the generation of a yellow-orange precipitate ions [37,38], which affect the aquatic and terrestrial biodiversity by seeping into groundwater as well as in surface and well waters and thus posing a threat to humans’ health [11,12,39,40]. Additionally, in mining, the tailings, which contain hazardous metals, such as cadmium (Cd), copper (Cu), chromium (Cr), lead (Pb), nickel (Ni), arsenic (As) and zinc (Zn), accumulate in soil and plants, as well as in surface water and groundwater [11,12]. Cui et al. [41] claimed that the co-exploitation of coal and uranium resources (depending on the geological conditions) can induce a feasible model concerning environmental damage, which occurs naturally during mining activities. Over time, uranium mining proved to be a strong challenge for the environmental protection authorities worldwide. Uranium, with its decay products resulting from mining activities, is stored in tailing dumps and has been over the years a source of hazard contaminants for the environment, and implicitly for humans [11,12,42,43,44,45]. 

**Figure 1:**
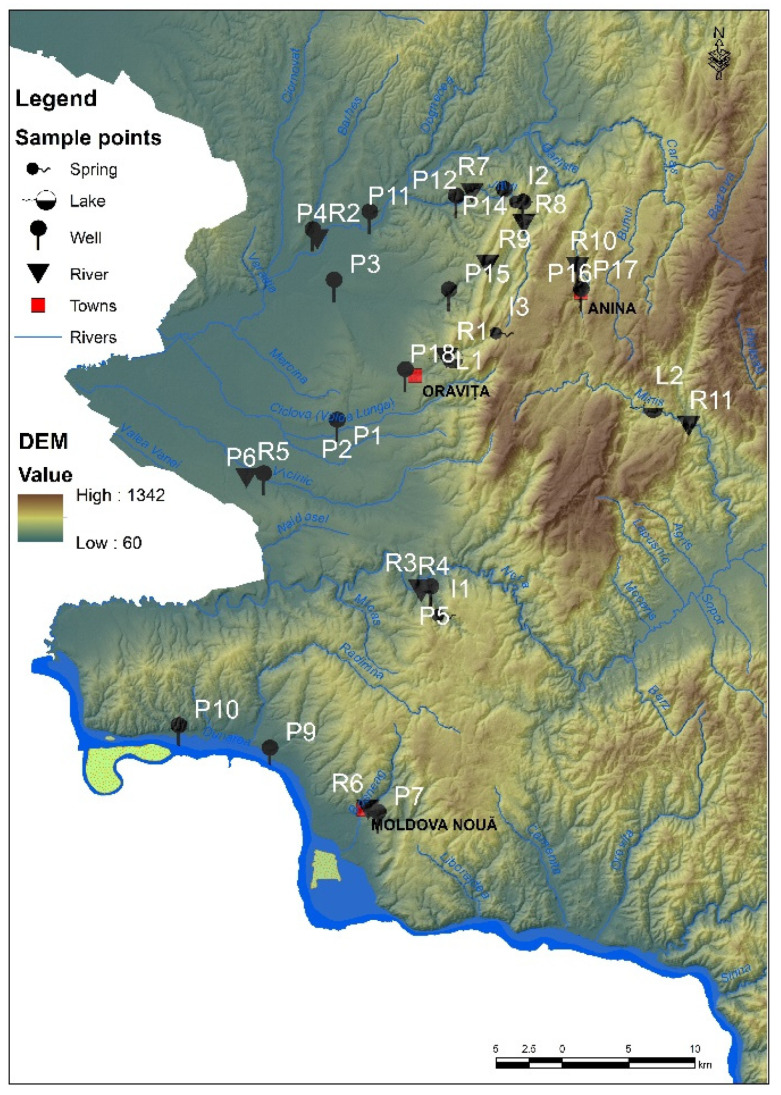
The southern area of the Banat Mountains, Romania (former mining area).

Major environmental problems are found in one of the most beautiful regions of Romania, namely Banat [11,12,46] (Figure 1), where uranium, copper and coal mines are largely closed, leaving behind tailing dumps, acid waters, destroyed soils, and heavily polluted waters, but also whole generations of people affected by occupational diseases. As mentioned in previous studies [11,12], uranium mining from both Ciudanovita and Lisava mines (Figure 1), which are located in the Banat Region, has left behind over time thousands of tons of tailings (approximately 30 tailing dumps), a real danger for the ecological balance of the area and, implicitly, for the inhabitants. In addition, Pehoiu et al. [11,12] revealed that, in terms of heavy metals and radioactivity due to mining activities, soil/sediment/plants pollution have led to a significant negative impact on human health, increasing the risk of intestinal, kidney, respiratory, heart, and tumor diseases, with a direct effect on the normal development of children.

The purpose of this study is the assessment of the quality of surface water and groundwater affected by mining activities, especially tailing dumps/mining landfills in the areas of closed uranium mines from Ciudanovita and Lisava, as well as of copper ore mining from Moldova Noua, and charcoal mines from Anina, Banat Region, Romania (Figure 1). So far, mining activities have led to the pollution of the hydrographic system with heavy metals, where concentrations proved to be well above Romanian legal limits. In this respect, in order to complete a multivariate analysis, the heavy metal concentration was correlated for the very first time with the values of physicochemical indicators of water (i.e., electrical conductivity, dissolved oxygen, pH, resistivity, salinity, and oxidation-reduction potential), so as to shape a regional based model on spatial distributions and the variability of toxic contaminants from the hydrographic basin of Banat, Romania, as a consequence of former mining exploitation. 

## 2. Materials and Methods

### 2.1. Site Description

Field surveys and water sampling were carried out in Romania, in the southern area of the Banat Mountains, within the perimeter of former mining exploitation. From a geological point of view, the mountainous area (1200 m in altitude with a fragmentation degree from 500 to 700 m) stands out through the existence of sedimentary deposits specific to the Getic Canvas, as well as through the Resita-Moldova Noua syncline, and in the hilly and depression areas, through suites of alluvial deposits—Mio-Pliocene—Quaternary Age (marls, clays). As a result of the differential erosion influenced by the presence of rocks with different hardness and also of the tectonics, a series of local depression areas were formed, such as Anina, Ciudanovita, and Lisava (Figure 1) [47,48,49].

The submountain depression of Oravita is situated in the south-western part of the low mountains of Banat. The depression is crossed from east to west by the Oravita brook, the Caras and Nera Rivers. Contact with the neighboring mountainous regions is well cut and often in steps, which explains the tectonic origin of this depression. It may be described as a more fragmented region to the east and south, where it is in direct contact with the mountain; furthermore, it may be stated that the degree of fragmentation decreases proportionally to the west. The average altitude is about 150–200 m, but on the east side it exceeds 300 m. The Caras River flows from north-east to south-west along the entire depression, determining its inclination line. It has a well-defined valley, due to the limestone relief, with a series of tributaries, more numerous on the left side. The Moldova Noua depression develops at the south-eastern foot of the Locva Mountains, on the left bank of the Danube, at an average altitude of 250–300 m. The hydrographic network that drains the area belongs to hydrographic basins from the southwestern part of the country: Caras, Nera, but also direct tributaries of the Danube on the southern slopes of the Locva Mountains—Almaj [50].

The Caras springs from the western slope of the Semenic Mountains from an altitude of 680 m, having a length of 79 km in the Romanian territory; it flows directly into the Danube in the territory of Serbia. The Caras River collects 31 tributaries. The Nera springs from the Semenic Mountains and flows into the Danube forming a 15 km state border with Serbia. The hydrostructures in the mountain area are concentrated in the upper precambrian crystalline schists, lower carboniferous crystalline limestones and dolomites, Jurassic age detrital and carbonate deposits, Jurassic-Cretaceous carbonate, and detrital deposits and in conglomerates, sandstones, limestones, and upper Cretaceous marl limestone. In the areas of the intramountain depressions they are in predominantly detrital and subordinate carbonate deposits, of Badenian age, but also in alluvial deposits (sands, gravels, silt, subordinated intercalations of marls and clays) of Quaternary age [50].

They may be cross-border in nature. Groundwater dynamics are much slower than surface water dynamics and the impact on the quantitative and qualitative status of groundwater bodies may exceed the natural recharge rate of the aquifer [51,52,53]. The most common sources of pollution that can lead to the qualitative deterioration of groundwater are the diffuse sources. Tailing ponds, and tailing dumps, affect groundwater through changes in quality due to pollutants that are entrained by runoff and then into surface water, or directly by infiltration into groundwater. The interdependence of groundwater bodies with surface water is generated by the supply of two types of ecosystems, depending on the depth of the piezometric level, but also the categories of land use. There is also a relevant interconnection between the degree of soil pollution, and the vegetation cover, with an impact on the health of the population [11,12,54]. The hydrographic network falls into the type of Southwestern Carpathian water regime with rainfall supply in which the underground contribution in this geographical region is between 30–38%, which results in the relationship between hydrostructures and surface waters. In conditions of prolonged drought, surface water feeds the aquifer underground [50,55]. 

The geographical locations of the sampling points were taken using a Global Positioning System (GPS) and the ArcGIS Pro mapping software, which provided real data for selected sites.

### 2.2. Sampling and Sample Preparation

In this study, a total of 408 water samples, divided as 34 water samples/day/week of each month of autumn of the year 2019 (i.e., 18 samples/day/week each from wells, 11 samples/day/week from three rivers and their tributaries, 2 samples/day/week from Oravita Lake, and Minis Lake, as well as 3 samples/day/week from three springs) were randomly collected, thus covering the entire area of the former mining area in the Banat Region, Romania (Figure 1). Samples were collected according to SR EN ISO 5667-3:2018, SR EN ISO 5667-6:2017/A11:2020, and SR ISO 5667-4:2020 from the administrative territory of three cities (Oravita, Anina and Moldova Noua), as well as 11 rural settlements of the same chosen area (Figure 1, Table 1).

The sampling focused primarily on the underground aquifer structures (i.e., wells). In this respect, it can be mentioned that the water wells samples, noted as P1–P18, were collected at different depths as follows: P1 at 8 m, P2 at 4.5 m, P3, P11 and P13 at 4 m, P4 and P10 at 7 m, P5 and P14 at 8 m, P6 at 4.2 m, P7 and P8 at 5.5 m, P9 at 6 m, P12 at 3.5 m, P15 and P18 at 3.0 m, P16 and P17 at 0.5 m. Approximately 90% of the old wells contain non-potable water (revealed by the information plates inserted on the wells). These wells are in an advanced state of clogging. In this situation, the water supply is made by capturing through the hydrophore, from depths of about 7–8 m (i.e., P1, P4, P5, P10, and P14, corresponding to schools), with the mention that laboratory tests were not performed by the authorities and there was limitation/prohibition of water consumption by students in the Ciudanovita area, P14, Figure 1. Wells that capture water from the upper part of the aquifer are naturally or anthropically clogged (household waste, waste), excepting P2, P3, P9, and P11. 

In addition, for a clear view image, samples from three spring sources (noted I1, I2, and I3) were collected, usually used as drinking water by children, mainly I1 near school and I2 representing a slope spring at 500 m by the entrance to Ciudanovita settlement, as well as I3, located 10 km from Oravita City on the interfluve between the cities of Oravita and Anina, used by tourists as well as by habitants. Therefore, it was revealed that there are no wells between the above-mentioned localities to be used for farming activities.

Regarding surface water, the samples were collected downstream of the former mining operations, from 11 hydrographic points (Figure 1) of the rivers Caras, R2, Nera, R3, Minis, and R11, along with their tributaries (R1, R4–R10). Leaves and forest debris (branches, twigs, and tree trunks) were found in the river water. Visually, a physical change in the color of the water was observed in the case of the Lisava River (reddish-rust color), which could be caused by the fact that it drains the tailing deposits from the perimeters of the former uranium exploitation. Water samples from two lakes (Oravita Lake and Minis Lake, coded L1 and L2, respectively) were taken from the middle area of each lake, in difficult conditions, in which the access to the dam area of L1 is forbidden, and the entrance area of L2 is inaccessible.

After sampling, everything was stored in sterile high-density polyethylene (HDPE) bottles of 1 L capacity, rinsed 2–3 times with water sample. Sampling and handling procedures were carried out following the Surface Water Sampling-Operation Procedure [56]. All samples were pre-labelled, refrigerated and transported at 4 °C to avoid contamination. Then, each one was filtered through 0.45-μm cellulose membranes and transferred into pre-washed HDPE bottles. For the actual investigations, were taken two vials of 120 mL from each water sample, with the mention that one was used for physicochemical indicator analysis and the other one for heavy metals determination by inductively coupled plasma mass spectrometry.

### 2.3. Analytical Techniques and Quality Assurance

The values of physicochemical indicators (electrical conductivity, dissolved oxygen, pH, resistivity, salinity, and oxidation-reduction potential) of water samples were obtained immediately after sampling (i.e., at the sampling site itself), using electroanalytical methods, as a subsequent determination would provide erroneous information, according to EN and ISO standard methods (Table 2 and Table 3). In order to determine the physicochemical parameters of water, the samples do not need preliminary preparation. The abovementioned physicochemical indicators were determined using WTW™ inoLab™ Multiparameter Digital Benchtop (WTW™ 1FD47K, Fisher Scientific, Leicestershire, UK).

Microwave-assisted pressure digestion was used for samples preparation. In this regard, the samples were mineralized in aqua regia (hydrochloric and nitric acids, in a ratio 3:1, according to SR EN ISO 15587-1:2003 standard method; high purity, Merck, Germany) using a TOPwave microwave-assisted pressure digester (Analytik Jena, Jena, Germany), with contactless real-time temperature and pressure monitoring for all TFM-PTFE vessels with samples, which can be removed individually after digestion. The water samples were acidified at pH < 2 before inductively coupled plasma mass spectrometry (ICP-MS) analysis. After digestion, the clear solutions were transferred with distilled water to 25 mL volumetric flasks. The concentration of metals, including Al, Cr, Mn, Fe, Ni, Co, Cu, Zn, Sr, Cd, and Pb, were determined using an iCAP™Q ICP-MS spectrometer (Thermo Fisher Scientific Inc., Waltham, MA, USA) in accordance with SR EN ISO 17294-2:2017 standard method (Table 2 and Table 3). The high sensitivity of this technique allows metals content determination to go up to ppb-ppt level (μg/kg or μg/L—ng/kg or ng/L), analyzing isotopes and perform multi-element determinations on a single sample. The analysis was performed in triplicate, in the standard mode (STD) for which the Qtegra Intelligent Scientific Data Solution software allowed the automatic correction of known isobaric interferences. The relative standard deviation (RSD) values were in the range of 0.01–2.66%.

Metals calibration curves showed good linearity over the concentration range (0.1 to 10.0 mg/L), with R^2^ correlation coefficients in the range of 0.990 to 0.999. The analytical curves for each analyzed elements were prepared using standard stock solutions (Certipur^®^, Certified Reference Material–ICP multi-element standard IV, Merck, Germany). The limits of detection (LODs) and limits of quantitation (LOQs) of the analyzed element were established using the calibration data and are presented in Table 4. Two standard reference materials (i.e., NIST SRM 1515 Apple leaves and SRM 2710a Montana I Soil) were used to verify the accuracy and traceability of the method. Recovery rates and analytical outputs of the reference materials were in the range of 95.3–101.7%. Accuracy and precision in the ranges of 91–104% and 1–9%, respectively, were considered good in terms of method performance characteristics.

### 2.4. Health Risk Assessment

Considering the equations presented and described in previous research [12], the following indexes were calculated:

- Estimated Daily Intake (EDI)

EDI = C_water_ I, (1)

where C_water_ = metal content in water, I = average intake rate: 2 L for adults and 1.5 L for children under the age of 12;

- Carcinogenic Risk (CR) associated to Pb exposure:

CR_Pb_ = EDI_Pb_⋯CSF_Pb_, (2)

where CSF_Pb_ = 0.0085 mg^−1^ kg day; acceptable risk levels for carcinogens range from 10^−4^ (risk of developing cancer over a human lifetime is 1 in 10,000) to 10^−6^ (risk of developing cancer over a human lifetime is 1 in 1,000,000);

- Daily Intake Metals

DIM = EDI/BM,(3)

where BM = body mass: 70 kg for adults and 14 kg for children;

- Health Risk Index (HRI),

HRI = DIM/RfD,(4)

where RfD = oral reference dose: 3 μg L^−1^ day^−1^ for Cr(VI), 1 mg L^−1^ day^−1^ for Cr(III), 140 μg L^−1^ day^−1^ for Mn, 20 μg L^−1^ day^−1^ for Ni, 40 μg L^−1^ day^−1^ for Cu, 300 μg L^−1^ day^−1^ for Zn, 1 μg L^−1^ day^−1^ for Cd, 35 μg L^−1^ day^−1^ for Pb,

(5) $$⇨\;\mathrm{HRI}=\frac{C_{\mathrm{water}}\;\cdot \;I}{\mathrm{BM}\;\cdot \;\mathrm{RfD}}$$

- Cumulative Health Risk (T_THQ_)

(6) $$T_{\mathrm{THQ}}=\overset{n}{\underset{i=1}{\sum }}{\mathrm{HRI}}_{i}.$$

### 2.5. GIS and Statistical Analysis

In this study, the software ArcGIS Pro developed by ESRI and Global Positioning System (GPS) helped develop the studied area maps and spatial interpolation of analyzed data. For the spatial interpolation, all data were integrated into GIS based on the inverse distance weighted (IDW) algorithm interpolation method (a geostatistical procedure) to visualize the quality indicators of drinking and surface waters, and their potential risk-prone areas. Statistical analysis was used to investigate the relationship between toxic metals between drinking water (wells and springs) and surface water (rivers and lakes), such as the main component analysis method (PCA) and Pearson correlation coefficient (PCC) using the IBM SPSS Statistics (v.21) software.

## 3. Results and Discussion

It is well known that surface water as well as groundwater frequently contains numerous chemical compounds provided by anthropogenic sources and various geochemical processes. In this respect, the assessment of physicochemical indicators regarding the quality of water is important for understanding the behavior of the toxic elements and their interrelationship in the chemical equilibrium of a particular geographical area. In spite of that, the threat to human health, in terms of water quality, are associated with: (1) pollution sources; (2) water treatability (the forming of disinfection by-products); and (3) direct health risks (faecal contamination, radioactive and, last but not least, nitrates and pesticides) [58]. As aforementioned, in the studied mining area (coal, uranium, copper), water, regardless of source (underground or surface), is mostly utilized for domestic and drinking purposes by the local population. Therefore, it is necessary to evaluate the suitability of water for human health concerns, knowing the fact that the chemical composition of water in terms of quality indicators changes over time and sometimes it is possible to reach a maximum threshold involving a water deterioration from Class II to Class III, or even exceed the alert threshold, by passing from Class III to Class IV. 

**Figure 2:**
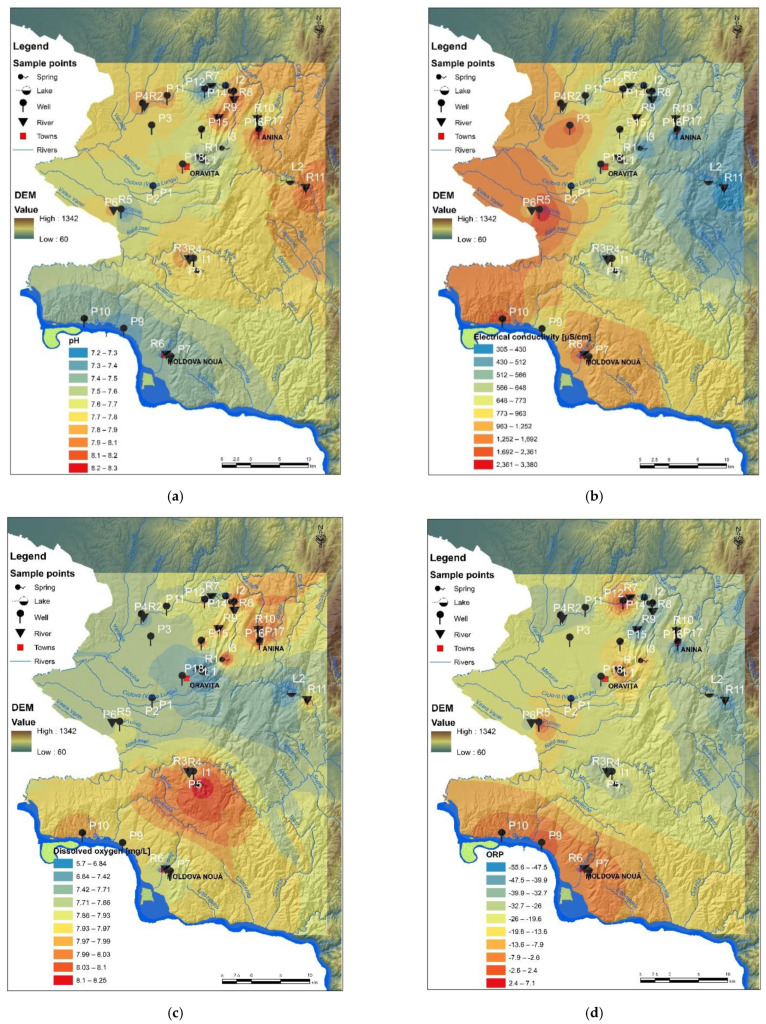

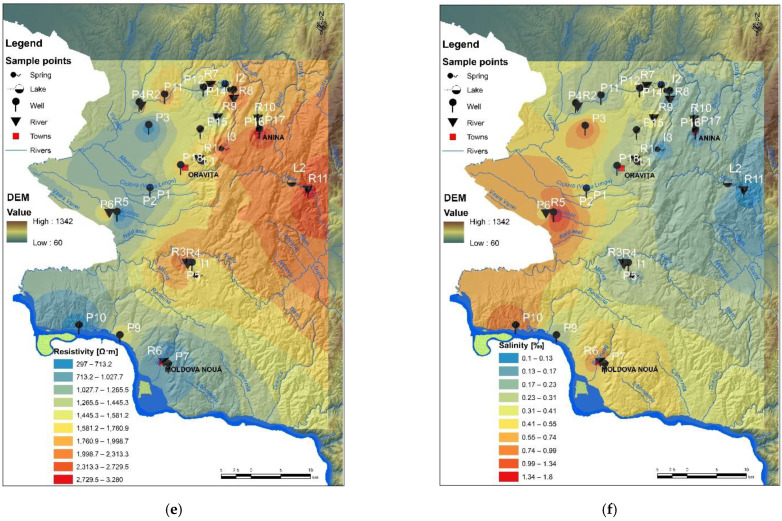
Physicochemical indicators of water quality: (**a**) pH; (**b**) electrical conductivity; (**c**) dissolved oxygen; (**d**) oxidation-reduction potential (ORP); (**e**) resistivity; and (**f**) salinity.

The pH value is considered a neutral indicator and also an important indicator when water hardness (alkaline water) is considered. According to the obtained data (Figure 2a), the pH is generally stable between 7.20 ± 0.31 and 8.20 ± 0.40, showing a weak alkaline water. Specifically, the collected sample from 18 water wells showed that the average of pH was 7.95 ± 0.45, except P7 (pH 7.35 ± 0.39), P9 (pH 7.21 ± 0.33), P10 (pH 7.23 ± 0.41), and P12 (pH 7.29 ± 0.52). This also means that the background value of well water quality is weakly alkaline. Therefore, the standard pH value for well water samples can be set to 8.00 ± 0.22. Analyzing the results, the water quality of wells from abandoned coal mines areas (Anina, Oravita) was affected in terms of drinking water indicators (i.e., pH, OD; Figure 2a,c), and further changes in heavy metal concentrations were expected. Regarding the pH average value of samples collected from three springs sources, I1, I2 and I3, it can be noted that the pH is different for each of them, such as 7.70 ± 0.29, 7.97 ± 0.32, and 7.39 ± 0.41, respectively.

In addition, the pH average value of surface water samples collected from 11 hydrographic points (Figure 2a) in Romania ranged between 7.30 ± 0.24 (R5) and 8.18 ± 0.41 (R11). On the other hand, the pH value for water samples of both lakes, L1 and L2, revealed the same weakly alkaline water, i.e., 7.41 ± 0.26 and 7.78 ± 0.37, respectively.

It is well known that the major cation and anion levels determine the electrical conductivity (EC) of water and determine the overall salinity [58]. Certainly, the conductivity and salinity levels indicate the contamination degree of water, especially when compared to benchmarks or reference values for expected natural concentrations of cations and anions (e.g., according to international/national legislation). The average EC of well water was higher in the south-eastern Banat Region (along the border with Serbia) that involves former and active copper mines (Figure 2b, Table 5). The highest average EC value was in the wells P6 (2.362 µS/cm). For samples P3, P7 and P10, from the abovementioned sites (Figure 2b, Table 5), the average EC values were 2092 µS/cm, 1378 µS/cm and 1692 µS/cm, respectively. The EC of water collected around of former uranium mines (Ciudanovita and Lisava), mainly corresponding to schools (children being directly exposed), ranged between 395 µS/cm (P14, P16) and 963 µS/cm (P12). Spring water samples I1, I2 and I3 have shown a lower EC value such as 541 µS/cm, 430 µS/cm, and 317 µS/cm. The correlation with the salinity results (Figure 2f) may be caused by an increase in ions for the copper mining area and EC from copper rocks and soil leaching in the rainy season of the autumn of year 2019. Both EC and salinity indicators (Figure 2b,f, Table 6) showed higher values for river water collected from the following points: R2 (773 µS/cm and 0.19%), R5 (851 µS/cm and 0.33), and R6 (1783 µS/cm and 0.71). It can be concluded that well waters and river waters, located around the copper mining area, contained various dissolved materials, along the ground and surface water flow path, resulted from mining activity and from other industrial activities specific to the zone. 

Oxidation-reduction potential (ORP) is one of the important indicators of redox-sensitive processes in water as well as in sediments. The negative values of ORP indicate an abundance of available electrons (Figure 2), therefore the drinking water with ORP negative and weakly alkaline pH can be considered an excellent antioxidant, with benefits for health (reduction of intestine inflammation, digestive pain, disease, and other symptoms caused by free radicals). In addition, many digestive problems are caused by a disturbance of the balance of beneficial microorganisms that live in the digestive tract and can be solved by supporting them [58]. “Friendly” bacteria in the intestines are 95% anaerobic therefore a negative ORP is needed. The consumption of water with negative ORP supports the development of beneficial bacteria in the intestinal tract and helps establish microbial balance. On the other hand, the spatial dynamics (i.e., between sediment-water and water-water vertical layers) and the temporal dynamics of ORP variations of surface waters (i.e., river, lake, spring) can be very important to overcome the limitations in the exchange process of minerals. The average ORP for Moldova Noua copper mining area, near the Danube, showed slightly negative to positive values throughout the monitoring campaign (Figure 2d,e). Furthermore, some wells (P7, P9, and P10) showed negative ORP values in September 2019 and then became positive in October and November 2019, due to the rainy period after recharge from rain infiltration that enhances the oxygen content. Furthermore, some water wells (P7, P9, and P10) showed negative ORP values in September 2019 and then became positive in October and November 2019, due to the rainy period; after wells recharge due to rainfall, the infiltration increases the oxygen content and pH of water decreases from 7.59 to 7.20 (Figure 2a). The positive indicator of water is given by dissolved oxygen (DO), which characterizes a healthy ecosystem. Generally, high levels of DO indicate a good quality of waters. According to Figure 2c, the samples collected around the Nera River (R3 and R4) as well as water samples collected from well P5 and spring I1 (in the south of Natural Reservation of Banat) showed higher DO levels. Nevertheless, the high temperature, as well as a high salinity level led to the decrease of the DO level in water. High productivity due to algae, cyanobacteria or floating vegetation decrease DO levels as plant material as cells die and are decomposed at the bottom of the lakes L1 and L2 (Figure 2c), consuming oxygen.

Water resources in mining areas from Romania, the Banat Region, are exposed to serious pollution. The contamination of surface and underground waters due to mining operations (e.g., uranium, copper, and charcoal) is strongly linked to the release of heavy metals. Some of these metals are considered naturally ubiquitous in surface waters and groundwater (sources for drinking water) and the impact on human health due to exposure to them is significant. In addition, the mobility of these elements increases in the case of mining activities due to the fact that contaminated water also passes through the cracks of rocks and mine drainage networks. On the other hand, the mining operations (uranium, copper, charcoal) generate huge quantities of poisonous solid residues (i.e., tailing dumps). The former copper and uranium mines, from Moldova Noua and Ciudanovita-Lisava, respectively, due to the drainage process, highly affect the groundwater and surface waters and further increase the levels of heavy metals [11,12]. In the frame of Romanian Regulation [57] the concentrations of Al, Fe, Cr, Ni, Co, Cu Zn, Sr, Cd, and Pb, vary depending on the water hardness as specified in five class categories (Table 3 and Table 4). In addition, Directive 2020/2184/EU and Directive 2013/39/EU [21,22] provided thresholds for cadmium and its compounds (Table 3 and Table 4) for the five water class categories (Class 1 to Class 5) in terms of hardness. On the other hand, Romanian and EU Regulations mention that natural background concentrations for metals and their compounds must be in compliance with the EQS values [22]. These values of metal concentrations depend on water hardness, as well as on other quality indicators, such as pH, electrical conductivity, dissolved oxygen, salinity, and oxidation-reduction potential, which led to the increase of metal bioavailability in the food chain, increasing the risk for human health. However, when compared with the WHO limits for drinking water [3], the Pb concentrations were up to 30 times higher than the limit, for sample P9, and this poses a real danger to humans, especially children. On the other hand, according to Order 161/2006 and Directive 2020/2184/EU, the waters of rivers and their tributaries can be classified into quality classes II, III or IV due to the high Pb content (10.58 ± 0.96–29.17 ± 1.31 μg/L) except for R6, R7, and R9. In addition, copper, nickel, and zinc concentrations are higher in samples collected around charcoal and copper mines. Compared with the values stipulated by Romanian and EU Regulations [22,57], the Cd concentrations, expressed as an average (Table 4), were higher than the allowed levels (two times higher, or even three times higher for surface water sample R2). Zhang et al. [59] claim that Sr concentration in drinking water higher than 0.3 µg/L provides a potential risk for human health. According to data revealed by Zhang et al. [59] and USEPA recommendation [60] regarding the maximum accepted level for strontium in water intended for human consumption, even if Romanian [61] and European Regulations [22] did not highlight a limit value, several water samples P3, P4, P5, P6, P10, and P13 showed a visible higher concentration of strontium.

Concentrations of Pb, Cu, Cd, Cr, and Ni were higher than national accepted values and the World Health Organization specifications [3] for water intended for human consumption in the case of all samples, with some exceptions for I3, R6, and R9 (Table 5, Appendix A). Chromium concentrations (Table 6, Appendix B) were the lowest in surface water samples (rivers and lakes). The concentration of toxic/carcinogenic metals in water samples decreases as follows: Pb > Cd > Ni > Cr.

**Figure 3:**
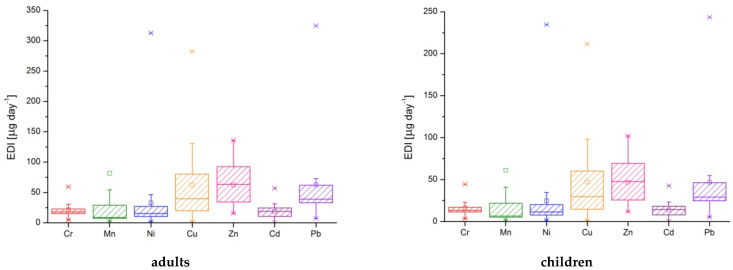
The estimated daily intake represented as box-plots for seven toxic metals in well water.

According to the Institute of Medicine [62] and the European Food Safety Authority [63,64], the values for estimated daily intake were higher than the recommended or tolerable levels, as follows (Table 7, Figure 3, and Table S1 from Supplementary Material):

- *chromium*: P2 and P10 for all categories (male, female, boys and girls 9–12 years); P3, P13, and P14 for female; P3 and P13 for girls 9–12 years;
- *cadmium*: P1, P2, and P10 for male and female; all samples except I1 and I2 for boys and girls 9–12 years;
- *lead*: P9 for male and female; P6–P9 for boys and girls 9–12 years;
- The lifetime risk of developing cancer due to lead exposure was beyond the imposed limits in the samples P8 and P9 (for all categories).

**Figure 4:**
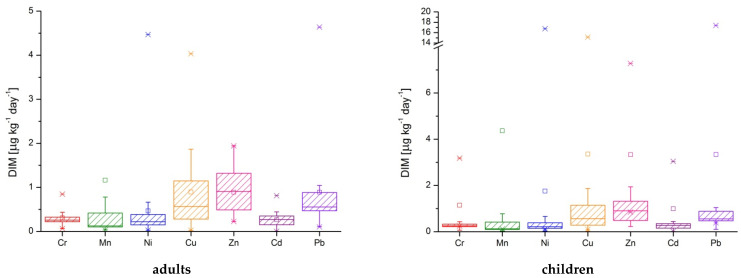
The daily intake metals represented as box-plots for seven toxic metals in well water.

Considering the obtained results for estimated daily intake and the body mass for adults (~70 kg) and children (~14 kg) the values for daily intake metals from drinking waters are presented in Table 8, Figure 4, and Table S1 from Supplementary Material. The maximum values for Cr, Mn, Ni, and Cu were determined in the P2 sample (for adults and children). In both cases, the highest value for Zn was determined in P18 sample, for Cd in P1 sample, respectively in P9 sample for Pb.

**Figure 5:**
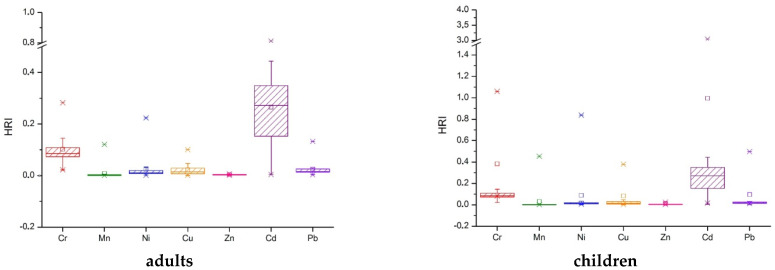
The health risk index represented as box-plots for seven toxic metals in well water.

The health risk index (HRI), also known as the target hazard quotient (THQ), represents the evaluation of health risks induced by the consumption of contaminated food and/or drinking water. It is used to estimate the potential health effects expected as a consequence of ingestion and to evaluate the adverse effects. If the HRI value is less than 1, then no adverse health effects are expected. However, this study takes into account only the metals from drinking water, not the entire content ingested daily. Data shown in Table 9, Figure 5, and Table S1 from Supplementary Material highlights that for adults, all drinking water samples are safe for health if the metals ingested from other sources are limited, but P1, P2, P7, P8, P10–P14, P16, and P18 samples are toxic for children.

Pearson correlation coefficient (PCC) and principal component analysis (PCA) are commonly used methodologies for the selection of linear variables [65]. The analysis of the connection between the analyzed metals from surface water and groundwater was carried out by calculating the Pearson coefficient in order to identify possible dependence relationships and associations between these variables. In addition, the Person coefficient, as a reliable coefficient of linear correlation, has been used to determine the strength of the association between two or more variables. For the significance testing, the correlation coefficient is used, taking values between −1 and +1, where −1 indicates a perfect negative linear relationship, 0 indicates no correlation, and 1 indicates a perfect positive linear relationship.

Table 10 shows the results of the Pearson correlation analysis. For toxic metals in drinking water (Table 10a), strong correlations (>0.50) are observed between Al and Cr, Al and Mn, Al and Fe, Al and Ni, Al and Cu, Cr and Mn, Cr and Fe, Cr and Ni, Mn and Fe, Mn and Ni, Mn and Cu, Fe and Ni, Fe and Cu, Ni and Cu. On the other hand, the correlations between toxic metals identified in surface water (Table 10b) are generally weaker than in drinking water, for example between Al and Cr, Cr and Mn, Cr and Mn, Cr and Fe, Cr and Cu. However, strong correlations can be observed between elements such as Co and Cu, Cr and Sr, Al and Cd, Mn and Cd, Fe and Cd in the case of surface waters; this may suggest that metals in surface waters may be influenced by different adjacent sources, including anthropogenic sources [66].

As already mentioned in previous studies by Pehoiu et al. [11,12], the study area is a mountainous region, partly in the category of protected area, according to Romanian legislation, so the high concentration of toxic metals is mainly due to old mining activities carried out over 60 years (i.e., uranium, copper, and coal mining), as well as anthropogenic activities, rather than being a consequence of the usual agricultural and/or industrial activities. The positive correlation between different heavy metals and light metals indicates the same sources. Heavy metals are considered to be the main pollutants of the environment, with a negative effect on the quality of ecosystems, as highlighted in a number of studies [11,12]. The historical and present pollution, due to the mining activities in the study area, led to the accumulation of heavy metals in soils, irreversibly degraded the soil quality, as well as the underground and surface water quality. The results of the study show that both natural and mining exploitation sources are responsible for the high concentration of various light and heavy metals in surface water and water intended for human consumption and daily activities.

The Principal Component Analysis (PCA), a data reduction technique that summarizes several variables to the essential components, was applied to determine the likely sources of heavy metals and their associations in drinking water (Table 11).

The principal component analysis was applied to 21 variables, representing the entire set of drinking water quality data from the analyzed sites. The PCA generated many principal components (PCs), but only those components with an eigenvalue > 1 were taken into account. The results of the analysis regarding the main components showed that the contribution of four components is responsible for 82.782% of the total variation of the data (Table 11). The first component of the PCA was responsible for 47.696% of the variations with an own value of 5.247. In terms of this component, metals such as Al (0.986), Cr (0.709), Mn (0.979), Fe (0.987), and Ni (0.988) have a strong charge; this can be explained by their presence in the Earth’s crust. On the other hand, these elements are a consequence of industrial activities, mainly mining extractions and smelting plants. The second component represented a variation of approximately 14.014% with its own value of only 1.542. Factor 2 had a strong positive charge for Co (0.704) and Zn (0.882); both elements have an anthropogenic origin and the main sources are the mining and industrial sites from the southwestern part of Romania [67]. The third component displayed a smaller variation than the first two components, namely 11.153%, with an eigenvalue of 1.227 dominated by the positive charge of Cr (0.273), Cu (0.239) and Cd (0.642) through copper and charcoal mining and a strongly negative charge in the case of Pb (−0.830) through the former uranium mines. Similarly, the fourth component of the PCA accounted for approximately 9.917% of the total variation with an eigenvalue of 1.091 and was dominated by elements from Earth’s crust: Cr (0.294), Co (0.219), and Sr (0.934) (Table 12).

In the case of surface water (Table 13), three main components explained 79.505% of the total variation of the data and obtained eigenvalues greater than 1, respectively: PC1 = 46.698% and an eigenvalue of 5.137; PC = 19.109% and a value of 2.102; and PC3 = 13.698% and an individual value of 1.507. Component one had Al, Mn, Fe, Ni, and Cd with high charge scores (>0.890); component two has a positive charge of Cr (0.738), Zn (0.753), Sr (0.539) and Pb (0.720); while in component three Co (0.862), Cu (0.801), and Pb (0.415) had high load scores.

Knowing the distribution of toxic metals both in surface and underground waters is an essential factor for the protection of the environment and human life. Water represents one of the primary sources of life and its importance must prevail over any argument against it when it comes to preserving it qualitatively as well as quantitatively; therefore, it is paramount for present and future generations to try to reach this desideratum. Identifying the toxic metal sources of pollution is important for strategy development and for further actions in order to prevent and fight pollution. This study reveals significant concentrations of toxic metals in water, which entails the need to find administrative solutions in environmental protection and alerting the population regarding the risks generated by daily intake. In addition, the statistical correlations were important in establishing the metal sources and relations between them.

## 4. Conclusions

This study is the third in a series of investigations conducted by the authors, and it is certainly the most comprehensive research regarding former uranium, copper, and charcoal mines from a particular geographical area of Romania. In this respect, the present scientific endeavor focused on two areas, which include former extraction uranium ore sites, Ciudanovita and Lisava, as well as copper ore from Moldova Noua and charcoal mines from Anina, Banat Region, Romania. It highlighted that, for the first time, the heavy metal concentration was correlated with the values of physicochemical indicators of water (i.e., EC, DO, pH, resistivity, salinity, and ORP) by using a multivariate analysis. The purpose was to shape a regional based model on spatial distributions and the variability of toxic contaminants from the hydrographic basin of Banat, Romania, as a consequence of former uranium, copper, and charcoal mines. Although the actions were less visible on a high scale and the studies barely existed, awareness regarding the risk should be present among the population. Therefore, it was mandatory for a complex study to be undertaken in order to assess the state of those areas and how much it will impact them from an anthropogenic point of view. In this regard, 11 metals including Al, Cr, Mn, Fe, Ni, Co, Cu, Zn, Sr, Cd, and Pb from different water samples (well, spring, river, and lake), were collected from three particular geographical mining areas, and investigated. 

Non-carcinogenic and carcinogenic health risks of seven heavy metals were assessed using the estimated daily intake (EDI), daily intake metals (DIM) and target hazard quotient (THQ). The obtained THQ values were within the acceptable limits for cancer risks for adults, but (as was highlighted) are toxic for children in the case of eight samples from 18. The HRI and THQ values for Cd and Pb for children were three times higher than those for adults. This is a source of concern as their prevalence in well water exposes children and residents in the Banat Region to the risk of various types of cancers. 

The data obtained were subjected to a multivariate analysis in order to investigate the correlation and emphasize the significant differences between metal concentrations and potential risks to human health (both adults and children). Therefore, for toxic metals in well water samples, strong correlations (>0.50) are observed between Al and Cr, Al and Mn, Al and Fe, Al and Ni, Al and Cu, Cr and Mn, Cr and Fe, Cr and Ni, Mn and Fe, Mn and Ni, Mn and Cu, Fe and Ni, Fe and Cu, Ni and Cu.

Pb was found to have a significant correlation with Cd, indicating that both metals originated from the same source. Concentrations of Pb, Cu, Cd, Cr, and Ni were higher than national allowed values and the World Health Organization specifications for water intended for human consumption for all water samples, except I3, R6, and R9. Chromium concentrations were the lowest in surface water samples (rivers and lakes). The level of toxic/carcinogenic metals including Pb, Cd, and Ni in water samples was higher than the WHO recommendations and represents a real risk for human health, especially for children.

The data provided by this research can be used to develop a framework for quantifying some of the physicochemical indicators that stakeholders and authorities use when preparing guidelines for water quality (GWQ), taking into consideration these mining areas, which cause strong pollution of the environment and pose a risk to human health.

## Figures and Tables

**Table 1:** Geographical coordinates of sampling sites—Global Positioning System (GPS).

Sample	Longitude E	Latitude N	Sample	Longitude E	Latitude N
P1	23.39832	39.38290	R1	24.21930	39.90256
P2	23.39833	39.38291	R2	23.24865	40.79496
P3	23.37480	40.43781	R3	24.01678	38.16085
P4	23.21433	40.81705	R4	24.03186	38.13913
P5	24.10365	38.12990	R5	22.71483	38.99809
P6	22.84391	38.98037	R6	23.63060	36.49909
P7	23.70610	36.43110	R7	24.41383	41.14350
P9	22.89166	36.91335	R8	24.80368	40.90972
P10	22.20329	37.08214	R9	24.53044	40.60111
P11	23.64375	40.95181	R10	25.21047	40.58597
P12	24.29443	41.07081	R11	26.04700	39.38808
P13	24.65663	41.12049	L1	24.21350	39.89641
P14	24.79810	41.02371	L2	25.72013	39.52551
P15	24.24095	40.36473			
P16	25.23702	40.36816			
P17	25.20822	40.55357			
P18	23.91186	39.76330			
I1	24.20205	37.96310			
I2	24.78435	41.07617			
I3	24.63909	40.08850			

**Table 2:** Physicochemical parameters for water intended for human consumption provided by both European and Romanian legislations, and standard methods.

	Physicochemical Parameters [Units]	European Standard Value *	Romanian Standard Value ****	Standard Method
Water quality intended for human consumption	Hydrogen ion concentration [pH unit]	≥6.5 and ≤9.5	6.5–8.5	SR EN ISO 10523:2012
	Electrical Conductivity @25 °C [μs/cm]	2500	2500	SR EN 27888:1997
	Dissolved Oxygen [mg/L]	5.0	9.0	SR EN ISO 5814:2013
	Aluminum (Al) [μg/L]	200	NA	SR EN ISO 17294-2:2017
	Chromium (Cr) [μg/L]	25 (50) **	25	SR EN ISO 17294-2:2017
	Manganese (Mn) [μg/L]	50	50	SR EN ISO 17294-2:2017
	Iron (Fe) [μg/L]	200	300	SR EN ISO 17294-2:2017
	Nickel (Ni) [μg/L]	20	10	SR EN ISO 17294-2:2017
	Cobalt (Co) [μg/L]	NA	10	SR EN ISO 17294-2:2017
	Copper (Cu) [μg/L]	2	20	SR EN ISO 17294-2:2017
	Zinc (Zn) [μg/L]	NA	100	SR EN ISO 17294-2:2017
	Strontium (Sr) [μg/L]	0.3	0.5	SR EN ISO 17294-2:2017
	Cadmium (Cd) [μg/L]	5	0.5	SR EN ISO 17294-2:2017
	Lead (Pb) [μg/L]	5 (10) ***	5	SR EN ISO 17294-2:2017

* [22]; ** Chromium (Cr)—The parametric value of 25 μg/L shall be met, at the latest, by 12 January 2036; the parametric value for chromium until that date shall be 50 μg/L [22]; *** Lead (Pb)—The parametric value of 5 μg/L shall be met, at the latest, by 12 January 2036; the parametric value for lead until that date shall be 10 μg/L [22]; **** Depending on water hardness classes of water (i.e., class I to V) provided by Romanian Order 161/2006 [57]; NA = not available.

**Table 3:** Physicochemical parameters for surface water (including rivers and lakes) provided by both European and Romanian legislations, and standard methods.

	Physicochemical Parameters [Units]	European Standard Value **	Romanian Standard Value *	Standard Method
Surface water quality	Hydrogen ion concentration [pH unit]		6.5–8.5	SR EN ISO 10523:2012
	Electrical Conductivity @25 °C [μs/cm]			SR EN 27888:1997
	Dissolved Oxygen [mg/L]		4.0–9.0	SR EN ISO 5814:2013
	Aluminum (Al) [μg/L]	1.6		SR EN ISO 17294-2:2017
	Chromium (Cr) ** [μg/L]		50–>250	SR EN ISO 17294-2:2017
	Manganese (Mn) [μg/L]		100–>1000	SR EN ISO 17294-2:2017
	Iron (Fe) [μg/L]		500–>1000	SR EN ISO 17294-2:2017
	Nickel (Ni) [μg/L]	4–13	25–>100	SR EN ISO 17294-2:2017
	Cobalt (Co) [μg/L]		20–>100	SR EN ISO 17294-2:2017
	Copper (Cu) [μg/L]		30–>250	SR EN ISO 17294-2:2017
	Zinc (Zn) [μg/L]		200–>1000	SR EN ISO 17294-2:2017
	Strontium (Sr) [μg/L]	NA	NA	SR EN ISO 17294-2:2017
	Cadmium (Cd) [μg/L]	≤0.45–1.5	1–>5	SR EN ISO 17294-2:2017
	Lead (Pb) [μg/L]	1.2–14	10–>50	SR EN ISO 17294-2:2017

* Depending on water hardness classes of water (i.e., class I to V) provided by Romanian Order 161/2006 [57]; ** Directive 2013/39/EU of the European Parliament and of the Council of 12 August 2013 amending Directives 2000/60/EC and 2008/105/EC as regards priority substances in the field of water policy [21]; NA = not available.

**Table 4:** LODs and LOQs of analyzed elements by ICP-MS technique.

Metals	LOD [μg/L]	LOQ [μg/L]
Al	7.04	14.38
Cr	5.47	11.98
Mn	2.70	6.95
Fe	4.43	8.07
Ni	4.58	10.41
Co	0.73	1.99
Cu	10.82	9.07
Zn	9.07	16.72
Sr	1.37	3.21
Cd	3.04	5.22
Pb	0.89	1.88
Al	7.04	14.38
Cr	5.47	11.98

**Table 5:** Mean concentration of metals (expressed as µg/L ± S.D.) in freshwaters (wells and springs) from the Banat mining area.

Sample	Al	Cr	Mn	Fe	Ni	Co	Cu	Zn	Sr	Cd	Pb
P1	298.49 ± 5.18	7.96 ± 0.37	6.86 ± 0.10	24.22 ± 0.83	15.31 ± 0.06	0.98 ± 0.02	94.96 ± 0.53 *	37.31 ± 0.86	334.66 ± 4.56	28.43 ± 1.08 *	9.04 ± 0.47
P2	6732.37 ± 16.96 *	29.68 ± 0.19 *	592.07 ± 5.49 *	2273.27 ± 29.75 *	156.42 ± 2.08 *	5.16 ± 0.08	141.19 ± 1.89 *	18.27 ± 1.05	355.22 ± 2.95	12.95 ± 1.06	30.93 ± 0.95
P3	73.52 ± 3.48	15.22 ± 0.30	4.76 ± 0.06	40.22 ± 0.38	12.80 ± 0.47	1.38 ± 0.02	36.82 ± 0.26	31.94 ± 1.42	928.87 ± 11.34	5.02 ± 0.52	18.72 ± 0.74
P4	803.55 ± 11.87 *	11.19 ± 0.20	27.30 ± 0.37	132.88 ± 0.57 *	23.19 ± 0.59	0.43 ± 0.06	42.55 ± 0.53	31.77 ± 1.45	820.02 ± 6.67	4.01 ± 0.38	16.95 ± 0.48
P5	103.47 ± 3.16	9.48 ± 0.74	3.52 ± 0.06	14.22 ± 0.56	7.64 ± 0.21	1.81 ± 0.01	5.75 ± 0.38	19.32 ± 1.20	949.23 ± 13.07	7.32 ± 1.04	8.14 ± 0.31
P6	130.83 ± 3.08	8.52 ± 0.78	109.71 ± 1.54 *	45.93 ± 1.87	13.79 ± 0.57	5.78 ± 0.23	27.78 ± 0.98	34.67 ± 1.57	1841.12 ± 14.52	4.36 ± 0.28	54.20 ± 1.85
P7	371.98 ± 2.97	11.08 ± 0.22	26.40 ± 0.24	103.23 ± 2.44 *	21.84 ± 0.21	4.66 ± 0.05	19.84 ± 0.63	17.09 ± 0.67	288.04 ± 7.82	9.50 ± 0.77	36.55 ± 0.71
P8	118.37 ± 2.78	8.94 ± 0.39	4.21 ± 0.06	21.04 ± 2.02	8.97 ± 0.49	7.57 ± 0.22	15.61 ± 0.40	46.19 ± 1.98	293.01 ± 4.28	12.27 ± 1.21	90.43 ± 2.09 *
P9	2.43 ± 1.50	5.71 ± 0.36	4.19 ± 0.14	11.18 ± 1.24	13.49 ± 0.41	0.58 ± 0.01	23.14 ± 0.46	12.25 ± 0.55	279.95 ± 3.14	5.35 ± 0.41	162.33 ± 1.94 *
P10	39.16 ± 1.83	25.31 ± 1.46 *	2.38 ± 0.11	6.01 ± 1.25	5.53 ± 0.17	1.38 ± 0.09	13.99 ± 0.03	7.89 ± 0.05	730.26 ± 18.02	15.55 ± 0.61	10.83 ± 0.65
P11	8.57 ± 1.20	11.38 ± 0.19	18.96 ± 0.17	11.98 ± 1.26	5.20 ± 0.07	0.55 ± 0.01	13.31 ± 0.32	8.62 ± 0.85	221.18 ± 2.98	11.21 ± 0.80	21.17 ± 1.03
P12	155.28 ± 0.41	5.61 ± 0.48	4.36 ± 0.05	2.37 ± 1.16	6.76 ± 0.14	0.63 ± 0.01	27.05 ± 0.21	48.49 ± 1.67	310.69 ± 7.33	12.22 ± 1.65	26.52 ± 0.56
P13	14.46 ± 0.55	14.90 ± 1.03	10.90 ± 0.23	45.91 ± 1.55	7.34 ± 0.36	1.18 ± 0.06	5.56 ± 0.27	40.77 ± 2.13	946.83 ± 18.09	11.90 ± 0.93	19.46 ± 0.43
P14	12.82 ± 1.05	12.74 ± 0.86	3.98 ± 0.03	14.13 ± 1.89	12.06 ± 0.30	0.68 ± 0.04	40.10 ± 0.22	8.18 ± 0.63	114.77 ± 3.19	9.77 ± 0.78	16.49 ± 0.48
P15	7.19 ± 3.47	10.47 ± 0.13	10.91 ± 0.15	29.08 ± 3.39	7.41 ± 0.45	0.52 ± 0.01	9.83 ± 0.18	20.16 ± 0.99	279.38 ± 3.72	6.62 ± 0.54	18.68 ± 0.50
P16	46.27 ± 2.47	6.53 ± 0.13	3.89 ± 0.07	2.62 ± 0.92	5.08 ± 0.12	1.39 ± 0.02	2.93 ± 0.15	64.57 ± 3.02	89.07 ± 1.61	10.69 ± 0.62	29.37 ± 0.94
P17	44.61 ± 0.98	7.69 ± 0.34	14.58 ± 0.15	3.39 ± 2.38	13.53 ± 0.35	0.72 ± 0.02	5.50 ± 0.32	11.17 ± 0.66	225.01 ± 3.51	7.82 ± 0.43	16.72 ± 0.92
P18	39.75 ± 1.46	8.58 ± 0.23	1.18 ± 0.17	8.63 ± 0.99	1.26 ± 0.47	5.49 ± 0.15	13.05 ± 0.57	67.96 ± 2.34 *	382.43 ± 5.09	12.24 ± 1.31	21.71 ± 0.82
I1	59.16 ± 2.47	2.31 ± 0.36	1.48 ± 0.03	11.69 ± 0.93	1.05 ± 0.09	5.35 ± 0.08	53.08 ± 2.14 *	47.86 ± 1.82	171.40 ± 1.68	0.15 ± 0.01	10.34 ± 0.34
I2	2.63 ± 0.29	3.18 ± 0.18	2.04 ± 0.13	6.17 ± 0.73	3.48 ± 0.07	0.77 ± 0.02	0.94 ± 0.01	58.96 ± 2.66	321.28 ± 4.41	1.54 ± 0.12	32.57 ± 0.95
I3	5.66 ± 2.51	8.43 ± 0.92	2.24 ± 0.13	3.25 ± 1.99	2.05 ± 0.23	0.22 ± 0.01	65.37 ± 2.22	20.20 ± 0.81	330.36 ± 2.30	6.22 ± 0.10	3.62 ± 0.06

* indicates the problematic values.

**Table 6:** Mean concentration of metals (expressed as µg/L ± S.D.) in surface waters (river and lake) from the Banat mining area.

Sample		Al	Cr	Mn	Fe	Ni	Co	Cu	Zn	Sr	Cd	Pb
R1		76.24 ± 0.83	8.19 ± 0.05	8.16 ± 0.08	7.92 ± 1.46	1.68 ± 0.09	1.13 ± 0.10	3.04 ± 0.14	40.14 ± 0.77 *	498.84 ± 3.02	7.81 ± 0.81 *	23.71 ± 0.89
R2		294.01 ± 2.77	7.50 ± 0.33	30.83 ± 0.26	21.43 ± 2.20	2.43 ± 0.04	0.55 ± 0.07	1.25 ± 0.59	69.70 ± 2.73 *	146.99 ± 2.91	16.71 ± 1.37 *	21.58 ± 1.79
R3		56.01 ± 0.79	4.99 ± 0.31	8.37 ± 0.16	36.23 ± 1.25	7.86 ± 0.27	0.93 ± 0.02	2.76 ± 0.04	35.29 ± 1.82 *	130.18 ± 1.77	2.61 ± 0.56	13.49 ± 0.45
R4		118.37 ± 3.43	11.60 ± 0.56	6.51 ± 0.24	32.52 ± 2.48	4.49 ± 0.46	1.80 ± 0.06	38.71 ± 1.95	72.81 ± 3.05 *	566.31 ± 4.09	10.35 ± 0.72 *	29.17 ± 1.31
R5		1287.14 ± 11.73	13.15 ± 0.68	7.86 ± 0.57	13.88 ± 3.81	2.85 ± 0.41	0.03 ± 0.01	3.81 ± 0.60	75.23 ± 1.74 *	271.58 ± 2.01	5.33 ± 0.99 *	27.64 ± 1.62
R6		581.81 ± 6.08	11.76 ± 0.91	65.76 ± 2.03	13.90 ± 0.59	14.51 ± 0.57	1.72 ± 0.10	0.53 ± 0.01	36.99 ± 1.43 *	509.35 ± 5.29	11.82 ± 1.37 *	1.25 ± 0.02
R7		22.340 ± 3.85	12.21 ± 0.31	4.93 ± 0.06	29.54 ± 1.44	15.92 ± 0.18	0.47 ± 0.02	5.67 ± 0.14	14.39 ± 0.77	239.99 ± 3.68	6.66 ± 0.38 *	4.83 ± 0.05
R8		56.12 ± 27.53	12.03 ± 0.12	0.26 ± 0.03	4.98 ± 0.82	4.97 ± 0.03	1.50 ± 0.02	4.49 ± 0.08	75.54 ± 1.27 *	264.45 ± 4.19	8.39 ± 0.63 *	28.02 ± 1.17
R9		33.35 ± 0.91	4.09 ± 0.20	12.29 ± 0.15	62.79 ± 1.25	5.42 ± 0.19	0.09 ± 0.01	43.31 ± 1.75	45.51 ± 1.18 *	317.34 ± 2.72	0.58 ± 0.02	3.52 ± 0.03
R10		55.51 ± 2.28	3.03 ± 0.16	2.74 ± 0.06	3.18 ± 1.74	3.50 ± 0.08	0.57 ± 0.01	4.26 ± 0.41	50.97 ± 2.28 *	171.27 ± 3.69	0.42 ± 0.01	10.58 ± 0.96
R11		410.78 ± 2.81	3.17 ± 0.17	44.43 ± 0.18	37.52 ± 1.61	12.29 ± 0.30	11.18 ± 0.14	44.07 ± 1.94	18.14 ± 0.43	142.38 ± 3.15	4.28 ± 0.51	23.18 ± 0.61
L1		2614.97 ± 14.37 *	9.78 ± 0.97	136.68 ± 2.80	743.20 ± 18.92	49.30 ± 0.38	4.99 ± 0.98	32.72 ± 6.78	27.04 ± 2.20 *	464.02 ± 8.44	163.35 ± 9.85 *	8.51 ± 1.04
L2		411.15 ± 4.68	3.23 ± 0.55	65.64 ± 0.16	22.09 ± 1.79	12.02 ± 0.57	0.25 ± 0.03	3.57 ± 0.14	42.45 ± 1.57 *	179.56 ± 2.97	0.05 ± 0.01	8.59 ± 0.23
QC ** Order 161/2006	Class I	NA ***	25	NA ***	300	10	NA ***	20	1	NA ***	0.5	5
	Class II		50		500	25		30	5		1	10
	Class III		100		1000	50		50	10		2	25
	Class IV		250		2000	100		100	25		5	50
	Class V		>250		>2000	>100		>100	>25		>5	>50
Directive 2020/2184/EU	Class 1	1600	NA ***	NA ***	NA ***	4–13	NA ***	NA ***	NA ***	NA ***	0.08–0.45	1.2–14
	Class 2										0.08–0.45	
	Class 3										0.09–0.60	
	Class 4										0.15–0.90	
	Class 5										0.25–1.50	

* Indicates the problematic values. ** QC indicates the quality classes according to Order 161/2006; *** NA—not available.

**Table 7:** Estimated daily intake values and recommended daily intake/tolerable intake level for adults and children, as well as carcinogenic risk induced by lead content (Cr_Pb).

EDI [μg Day^−1^]	Cr	Mn	Ni	Cu	Zn	Cd	Pb	Cr_Pb
**Adults**								
min	4.62	2.36	2.10	1.88	15.78	0.30	7.24	6.154·10^−5^
max	59.36	1184.14	312.84	282.38	135.92	56.86	324.66	2.760·10^−3^
average	21.42	81.52	32.78	62.70	62.25	18.58	62.36	5.301·10^−4^
median	17.88	8.72	15.28	39.68	63.54	19.00	38.92	3.308·10^−4^
SD	13.02	256.96	65.32	68.70	38.14	11.92	70.98	6.033·10^−4^
**Children**								
min	3.47	1.77	1.58	1.41	11.84	0.23	5.43	4.616·10^−5^
max	44.52	888.11	234.63	211.79	101.94	42.65	243.50	2.070·10^−3^
average	16.07	61.14	24.59	47.03	46.69	13.94	46.77	3.975·10^−4^
median	13.41	6.54	11.46	29.76	47.66	14.25	29.19	2.481·10^−4^
SD	9.76	192.72	48.99	51.52	28.60	8.94	53.24	4.525·10^−4^
Recommended daily intake */Tolerable intake level ** [μg day^−1^]								
male	33 *	2300 *	1000 **	900 *	11,000 *	25 **	250 **	10^−6^÷10^−4^
female	25 *	1800 *	1000 **	900 *	8000 *	25 **	250 **	10^−6^÷10^−4^
boys (9–12 years)	25 *	1900 *	600 **	540 *	7000 *	5 **	50 **	10^−6^÷10^−4^
girls (9–12 years)	21 *	1600 *	600 **	540 *	7000 *	5 **	50 **	10^−6^÷10^−4^

**Table 8:** Daily intake metals from well water.

DIM [μg kg^−1^ day^−1^]	Cr	Mn	Ni	Cu	Zn	Cd	Pb
**Adults**							
min	0.066	0.034	0.030	0.027	0.225	0.004	0.103
max	0.848	16.916	4.469	4.034	1.942	0.812	4.638
average	0.306	1.165	0.468	0.896	0.889	0.265	0.891
median	0.255	0.125	0.218	0.567	0.908	0.271	0.556
SD	0.186	3.671	0.933	0.981	0.545	0.170	1.014
**Children**							
min	0.248	0.126	0.113	0.101	0.845	0.016	0.388
max	3.180	63.436	16.759	15.128	7.281	3.046	17.393
average	1.148	4.367	1.756	3.359	3.335	0.996	3.341
median	0.958	0.467	0.819	2.126	3.404	1.018	2.085
SD	0.697	13.766	3.499	3.680	2.043	0.638	3.803

**Table 9:** Health risk index (HRI) values of seven metals in well water samples, as well as the cumulative health risk (T_THQ_).

HRI	Cr	Mn	Ni	Cu	Zn	Cd	Pb	T_THQ_
**Adults**								
min	0.022	0.000	0.002	0.001	0.001	0.004	0.003	0.079
max	0.283	0.121	0.223	0.101	0.006	0.812	0.133	1.125
average	0.102	0.008	0.023	0.022	0.003	0.265	0.025	0.450
median	0.085	0.001	0.011	0.014	0.003	0.271	0.016	0.408
SD	0.062	0.026	0.047	0.025	0.002	0.170	0.029	0.245
**Children**								
min	0.083	0.001	0.006	0.003	0.003	0.016	0.011	0.296
max	1.060 *	0.453	0.838	0.378	0.024	3.046 *	0.497	4.218
average	0.383	0.031	0.088	0.084	0.011	0.996	0.095	1.688
median	0.319	0.003	0.041	0.053	0.011	1.018 *	0.060	1.530
SD	0.232	0.098	0.175	0.092	0.007	0.638	0.109	0.918

* Indicates the problematic values.

**Table 10:** (**a**) Pearson’s correlation matrix for toxic metal concentrations (drinking water); (**b**) Pearson’s correlation matrix for toxic metal concentrations (surface water).

(**a**)												
		**Al**	**Cr**	**Mn**	**Fe**	**Ni**	**Co**	**Cu**	**Zn**	**Sr**	**Cd**	**Pb**
Water intended for human consumption	Al	1										
	Cr	0.665 **	1									
	Mn	0.981 **	0.656 **	1								
	Fe	0.997 **	0.676 **	0.986 **	1							
	Ni	0.992 **	0.677 **	0.979 **	0.990 **	1						
	Co	0.290	0.083	0.342	0.296	0.276	1					
	Cu	0.755 **	0.429	0.720 **	0.737 **	0.746 **	0.168	1				
	Zn	−0.151	−0.450 *	−0.167	−0.161	−0.220	0.316	−0.159	1			
	Sr	−0.047	0.190	0.061	−0.048	−0.025	0.167	−0.084	−0.020	1		
	Cd	0.148	0.319	0.105	0.131	0.154	−0.023	0.318	−0.026	−0.165	1	
	Pb	−0.015	−0.183	0.019	−0.004	0.037	0.212	−0.126	−0.045	−0.029	−0.145	1
(**b**)												
		**Al**	**Cr**	**Mn**	**Fe**	**Ni**	**Co**	**Cu**	**Zn**	**Sr**	**Cd**	**Pb**
Surface water, including rivers and lakes	Al	1										
	Cr	0.265	1									
	Mn	0.803 **	−0.040	1								
	Fe	0.867 **	0.104	0.809 **	1							
	Ni	0.811 **	0.122	0.875 **	0.928 **	1						
	Co	0.286	−0.226	0.410	0.313	0.391	1					
	Cu	0.195	−0.212	0.230	0.365	0.296	0.590 *	1				
	Zn	−0.121	0.312	−0.396	−0.300	−0.527	−0.457	−0.193	1			
	Sr	0.275	0.543	0.239	0.306	0.252	−0.054	0.237	0.084	1		
	Cd	0.878 **	0.197	0.810 **	0.988 **	0.912 **	0.295	0.285	−0.237	0.351	1	
	Pb	−0.090	0.256	−0.389	−0.235	−422	0.175	0.046	0.617 *	0.024	−0.175	1

* Correlation is significant at the 0.05 level (2-tailed); ** Correlation is significant at the 0.01 level (2-tailed).

**Table 11:** Results of principal component analysis—total variance explained.

Component	Initial Eigenvalues			Extraction Sums of Squared Loadings			Rotation Sums of Squared Loadings		
	Total	% of Variance	Cumulative %	Total	% of Variance	Cumulative %	Total	% of Variance	Cumulative %
1	5.247	47.696	47.696	5.247	47.696	47.696	5.211	47.375	47.375
2	1.542	14.016	61.712	1.542	14.016	61.712	1.410	12.821	60.196
3	1.227	11.153	72.865	1.227	11.153	72.865	1.299	11.806	72.002
4	1.091	9.917	82.782	1.091	9.917	82.782	1.186	10.780	82.782
5	0.849	7.716	90.498						
6	0.477	4.340	94.838						
7	0.411	3.740	98.578						
8	0.136	1.239	99.818						
9	0.015	0.133	99.950						
10	0.004	0.037	99.988						
11	0.001	0.012	100.000						
Extraction Method: Principal Component Analysis									

**Table 12:** Rotational component matrix (Varimax with Kaiser normalization) for experimental variables in the drinking water samples (N = 21).

Element	Component			
	PC1	PC2	PC3	PC4
Al	0.986	0.029	0.018	−0.027
Cr	0.709	−0.358	0.273	0.294
Mn	0.979	0.043	−0.035	0.077
Fe	0.987	0.020	−0.001	−0.019
Ni	0.988	−0.028	−0.023	−0.015
Co	0.344	0.704	−0.229	0.219
Cu	0.786	0.023	0.239	−0.171
Zn	−0.224	0.882	0.112	−0.084
Sr	−0.012	0.045	−0.011	0.934
Cd	0.195	−0.003	0.642	−0.318
Pb	0.038	0.043	−0.830	−0.187
Extraction Method: Principal Component Analysis. Rotation Method: Varimax with Kaiser normalization ^a^				

^a^ Rotation converged in 5 iterations.

**Table 13:** Rotational component matrix (Varimax with Kaiser normalization) for experimental variables in the surface water samples (N = 13).

Element	Component		
	PC1	PC2	PC3
Al	0.890	0.165	0.122
Cr	0.266	0.738	−0.324
Mn	0.880	−0.211	0.148
Fe	0.940	−0.005	0.193
Ni	0.954	−0.193	0.131
Co	0.235	−0.189	0.862
Cu	0.211	−0.016	0.801
Zn	−0.353	0.753	−0.152
Sr	0.428	0.539	−0.046
Cd	0.948	0.084	0.642
Pb	−0.371	0.720	−0.830
Extraction Method: Principal Component Analysis. Rotation Method: Varimax with Kaiser normalization ^a^			

^a^ Rotation converged in 5 iterations.

## Data Availability

The data reported in this study are available on request from the first author.

## Supplementary Materials

The following supporting information can be downloaded at: https://www.mdpi.com/article/10.3390/ijerph192214866/s1.
